# Evaluation of cfDNA as an early detection assay for dense tissue breast cancer

**DOI:** 10.1038/s41598-022-12457-1

**Published:** 2022-05-19

**Authors:** Mouadh Barbirou, Amanda A. Miller, Erik Gafni, Amel Mezlini, Asma Zidi, Nathan Boley, Peter J. Tonellato

**Affiliations:** 1 Department of Health Management and Informatics, Center for Biomedical Informatics, School of Medicine, University of Missouri, 1 Hospital Drive, MA213, Columbia, MO 65212 USA; 2 Ravel Biotechnology Inc, San Francisco, CA USA; 3 Medical Oncology Division, Salah Azeiz Oncology Institute, University of Tunis El Manar, Tunis, Tunisia; 4 Medical School of Tunis, University of Tunis El Manar, Tunis, Tunisia

**Keywords:** Cancer, Genetics, Molecular biology, Biomarkers, Molecular medicine, Oncology

## Abstract

A cell-free DNA (cfDNA) assay would be a promising approach to early cancer diagnosis, especially for patients with dense tissues. Consistent cfDNA signatures have been observed for many carcinogens. Recently, investigations of cfDNA as a reliable early detection bioassay have presented a powerful opportunity for detecting dense tissue screening complications early. We performed a prospective study to evaluate the potential of characterizing cfDNA as a central element in the early detection of dense tissue breast cancer (BC). Plasma samples were collected from 32 consenting subjects with dense tissue and positive mammograms, 20 with positive biopsies and 12 with negative biopsies. After screening and before biopsy, cfDNA was extracted, and whole-genome next-generation sequencing (NGS) was performed on all samples. Copy number alteration (CNA) and single nucleotide polymorphism (SNP)/insertion/deletion (Indel) analyses were performed to characterize cfDNA. In the positive-positive subjects (cases), a total of 5 CNAs overlapped with 5 previously reported BC-related oncogenes (KSR2, MAP2K4, MSI2, CANT1 and MSI2). In addition, 1 SNP was detected in KMT2C, a BC oncogene, and 9 others were detected in or near 10 genes (SERAC1, DAGLB, MACF1, NVL, FBXW4, FANK1, KCTD4, CAVIN1; ATP6V0A1 and ZBTB20-AS1) previously associated with non-BC cancers. For the positive–negative subjects (screening), 3 CNAs were detected in BC genes (ACVR2A, CUL3 and PIK3R1), and 5 SNPs were identified in 6 non-BC cancer genes (SNIP1, TBC1D10B, PANK1, PRKCA and RUNX2; SUPT3H). This study presents evidence of the potential of using cfDNA somatic variants as dense tissue BC biomarkers from a noninvasive liquid bioassay for early cancer detection.

## Introduction

Breast cancer (BC) is the most prevalent cancer worldwide, with an estimated 2.3 million new cases in 2020^1^. According to the GLOBOCAN Cancer Tomorrow Prediction, incidences are expected to increase by 33.8% by 2040, suggesting a staggering 3 million new cases^2^. The incidence of mortality due to BC remains high in low-income countries due in part to the noticeable lack of options for early detection and therapy management^3^. In Tunisia, approximately 32.2 incident cases and 10.3 related deaths per 100.000 women were reported in late 2019^4^. Currently, mammography is the only noninvasive method for detecting evidence of possible BC in dense tissue patients, and ultrasound-assisted core needle biopsy is the only robust and effective means of obtaining definitive diagnosis and staging of BC. Together, they provide a tenuous tandem method for accurately detecting early BC in dense tissue patients. Mammography has low sensitivity, with up to 34% false negative diagnoses for female dense tissue patients under 40^5,6^. Complementary invasive ultrasound-assisted core needle biopsy has a number of shortcomings, including difficulty in targeting small lesions and the ability to miss underestimated lesions^7^. In addition, the mammography-tissue biopsy tandem does not provide detailed information (such as genetic mutations) that could be of great value in obtaining a precise diagnosis and delivering optimized therapy^7^. Collectively, these limitations suggest the untapped value of a more refined, robust, information-rich, noninvasive approach that reduces the need for repeated biopsies, unnecessary surgeries, and nonideally treatments, especially for women with dense breast tissue. In this context, liquid biopsy based on a simple noninvasive blood test is a very promising approach for investigating the tumor-derived material circulating in the bloodstream shed from primary tumors and their metastatic sites^8^. Among the tumor components in bodily fluids identified during the past decade, increasing attention has been given to circulating tumor DNA (ctDNA), which is now considered useful for the early detection and management of solid tumors such as those of colorectal, prostate and lung cancers^9^. The small nucleic acid fragments known as ctDNA (approximately 134–144 bp) are associated with abnormal cell structures and altered mechanisms^10^. Prior investigations have largely shown a high concordance between the ctDNA molecular profile and traditional tumor tissue using the same testing protocols^11^. Advances in next-generation sequencing (NGS) have simplified and improved the speed of the molecular identification and testing of ctDNA genomic alterations, proving value for novel target variant identification with the potential to improve patient outcomes^12^. Molecular investigations have demonstrated that the BC patient genome include somatic mutations and copy number alterations (CNAs) that correlate with cancer susceptibility and staging^13^. These genetic alterations can be detected in ctDNA from BC patients and thus are candidates for early BC detection and improved screening programs^14^. However, there are limited data regarding the variant profile differences among dense tissue subjects with positive mammograms and positive ultrasound biopsy versus those with positive mammograms and negative ultrasound biopsy against ctDNA molecular testing. In this study, we aimed to assess the differences in somatic variant profiles, including CNAs), single nucleotide polymorphisms (SNPs), and insertions/deletions (Indels), between subjects with positive mammograms and positive biopsies (pos-pos) versus subjects with positive mammograms and negative biopsies (pos-neg) using a ctDNA assay and to examine the differences in BC early detection and clinical outcomes of ctDNA testing.

## Methods

### Cohort

A cohort of 32 subjects with dense tissue and positive mammograms from Salah Azaiz Institute in Tunisia between June 2019 and January 2020 was recruited into the study. Clinical information was obtained through the medical records and a personal interview during sample collection. Cell-free DNA (cfDNA) sample collection was conducted after a positive mammogram but before ultrasound-assisted core needle biopsy. Microbiopsy test results were documented after confirmation by two independent physicians (radiologist and oncologist). This research was conducted through an Institutional Review Board-approved protocol (ISA/2019/04), and all subjects provided written informed consent for our study.

### Sample preparation and cfDNA sequencing

Ten milliliters of peripheral blood samples were obtained immediately before ultrasound-guided core needle biopsy. Plasma from Streck BCT tubes was prepared within 2 h after blood collection and stored at − 20 °C in the clinic until shipment to the research laboratory. cfDNA was isolated from 5 ml of plasma with a MagMAX Cell-Free DNA Isolation Kit (MM; Applied Biosystems, Thermo Fisher Scientific, Foster City, CA, USA) and then eluted in 60 µl of elution buffer according to the manufacturer’s protocol. cfDNA was quantified using a QuantiFluor dsDNA System and GloMax Discover Microplate Reader (Promega, Madison, WI, USA). The distribution of fragment lengths was checked by electrophoresis on an Agilent 2100 Bioanalyzer with a High Sensitivity Large Fragment 50 kb DNA Kit (Agilent, Technologies Inc., Santa Clara, CA, USA). An NEBNext Ultra II DNA Library Prep kit (New England Biolabs, UK; E7645) was used for cfDNA whole-genome library preparation. Higher-pass whole-genome sequencing was started with 10 ng of cfDNA input (median of 5 ng). Finally, 32 libraries were pooled and sequenced using 150 bp pair-end run reads and 8 bp dual-indices on an Illumina NovaSeq machine (Illumina, San Diego, CA, USA), producing cfDNA whole-genome sequences for each subject.

### Pathologic assessment and subject segregation

Pathologic tissues obtained by ultrasound-guided biopsy and under mammography for the whole cohort were reviewed by designated breast pathologists from Salah Azaiz Institute in Tunisia. According to the evaluation results from standard histology and mammogram imaging, the cohort was classified into two groups: the screening group, corresponding to subjects with positive mammography and negative biopsy (pos-neg; N = 12) and the cases group, corresponding to subjects with positive mammography and positive biopsy (pos-neg; N = 20). The absence of tumoral tissue as confirmed by examination was designated a “negative” biopsy, and a designation of a “positive” biopsy was made if the sample indicated stage I or II breast malignancy according to the 8th Edition of the American Joint Committee on Cancer (AJCC) Staging Manual for breast cancer^15^.

### cfDNA sequence analysis

**Figure 1:**
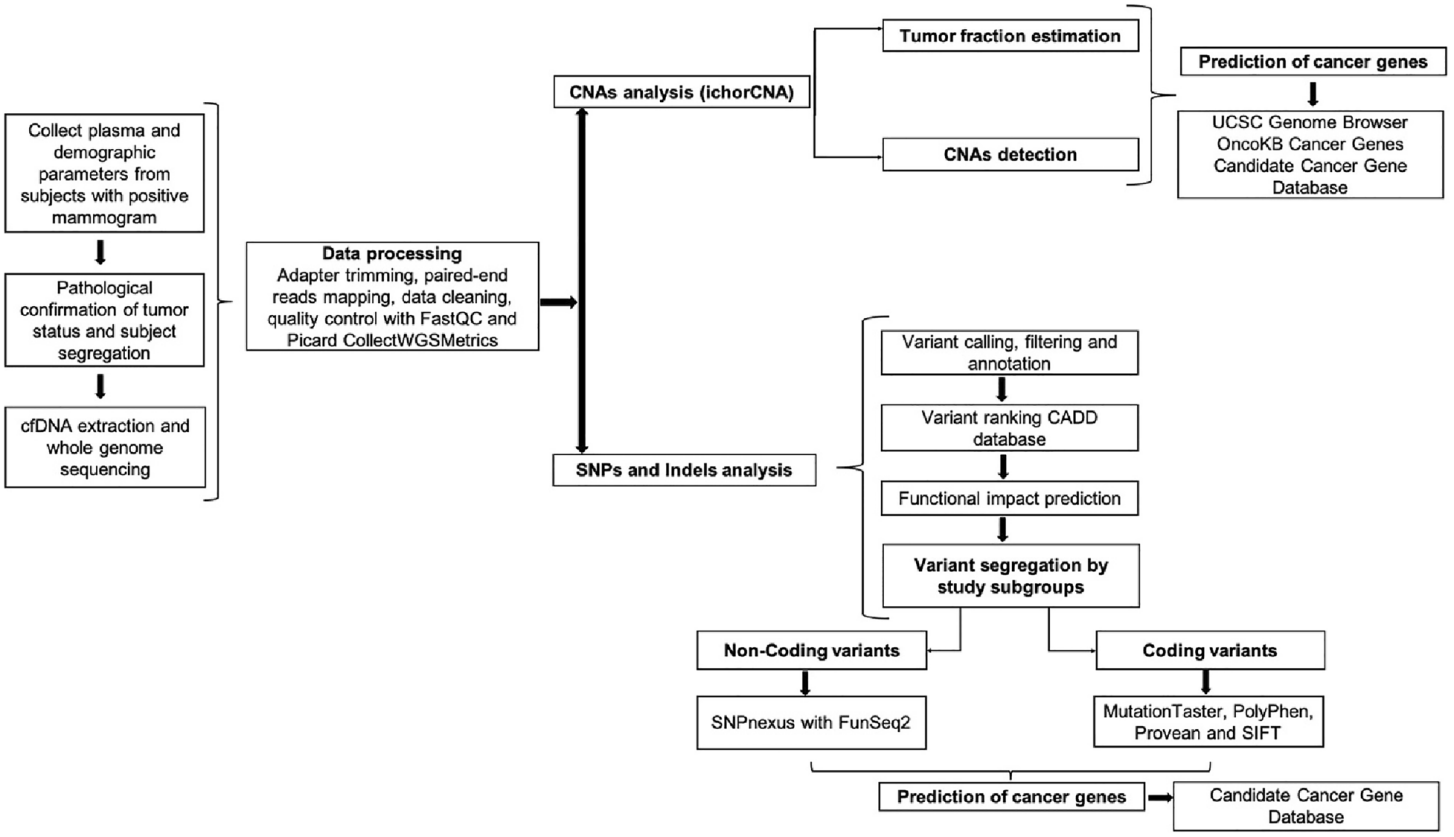
Schematic representation of the analysis workflow. *cfDNA* cell-free DNA, *QC* quality control, *CNA* copy number alterations, *SNPs* single nucleotide polymorphisms, *Indels* Insertion/deletions, *CADD* combined annotation dependent depletion, *UCSC* University of California Santa Cruz.

The analysis workflow performed in this study is summarized in Fig. 1. First, cfDNA whole-genome sequencing data were stored in Fastq files and then adapter trimmed using fastp (version 0.19.10) with default settings and -p-detect_adapter_for_pe^16^. The paired-end reads were aligned with BWA (version 0.7.17-r1188)^17^ to the GRCh38 human reference genome. The resulting BAM files were processed using the Picard (version 2.18.9) UmiAwareMarkDuplicatesWithMateCigar function (http://broadinstitute.github.io/picard/) to remove duplicate reads. FastQC (version 0.11.9) was run before and after adapter trimming to impose Fastq record quality control^18^, and Picard CollectWGSMetrics was used for BAM file quality control (http://broadinstitute.github.io/picard/).

### CNA

ichorCNA (version 0.3.2, https://github.com/broadinstitute/ichorCNA) was then applied to all high-quality aligned reads for each subject’s BAM files to estimate the tumor-derived DNA fraction (TF) and detect CNAs using all recommended default parameters except parameter adjustment to account for low cfDNA content samples^19^. Given the absence of an established control reference CNA set for these samples, no false-positive filtering was performed. Subsequently, the detected CNAs were grouped by subject status into “pos-pos” and “pos-neg” groups. The CNAs collected for each group were filtered to include only those shared by at least 2 subjects in the group and thereafter filtered to include alterations exclusive to that same group. These pos-pos and pos-neg exclusive CNAs were separately tested to determine the genes with which they overlapped using the UCSC Genome Browser^20^. The CNA-tagged genes were then tested against the Cancer Genes set found in the Precision Oncology Knowledge Base (OncoKB, 27) to determine which cancers (if any) the genes were associated with. These CNA-tagged cancer genes were then tested against the Candidate Cancer Gene Database^21^ to identify predicted associated cancers.

### SNPs and indels

Grouped by pathology type (pos-pos; pos-neg), each subject’s BAM files were then analyzed by the Mutect2 part of GATK (v. 4.1.8.1)^22^ to detect somatic SNPs and Indels within the 22 autosomes against a ‘panel of normals’ created from the 1000 Genomes project^23^ and the gnomAD^24^ database as a ‘germline-resource’ included in the GATK resource bundle (https://console.cloud.google.com/storage/browser/genomics-public-data/resources/broad/hg38/v0). Identified variants were then filtered using GATK FilterMutectCalls^22^ using the recommended default parameters and thereafter annotated using ANNOVAR^25^. Variants with a minor allele frequency (MAF) >  = 1% in the 1000 Genomes and ExAC databases were excluded^26^. Subsequently, candidate variants without a predicted deleterious nature were removed from consideration. To detect deleterious mutations, all variants were ranked using the CADD database (version 1.6), and those with a PHRED scaled score of > 10 were considered as having a probable deleterious function and retained in their respective pos-pos and pos-neg grouped collection^27^. For coding variants, the deleterious nature was predicted by MutationTaster^28^, PolyPhen V2^29^, Provean^30^, and SIFT^31^, provided by the dbNSFP database (version 4.1)^32^. The grouped variants predicted to be deleterious by at least three of the four prediction engines were retained. For noncoding variants, the designation of ‘deleterious’ was assigned after application of SNPNexus^33^ and a threshold of FunSeq2 score >  = 1.5^34^. The coding and noncoding deleterious variants were then collected into the pos-pos and pos-neg groupings. As with the candidate cfDNA CNAs, candidate cfDNA SNPs and Indels were filtered to include those appearing in at least two individuals within the group and thereafter exclusive to either pos-pos or pos-neg groups. These pos-pos and pos-neg exclusive variants were then used to identify their associated genes and the subsequent determination of cancer association using the Candidate Cancer Gene Database^21^.

### Statistical analysis

Statistical analysis was performed with R (version 3.6.2)^35^. Continuous variables are expressed as the means ± SDs, while categorical data are expressed as percentages of the total. Independent sample t tests were applied for intergroup comparisons of normally distributed continuous data, and chi-square tests were applied for categorical variables. P < 0.05 was considered statistically significant. The tumor fraction estimation boxplots of groups were created with the R-ggplot2 package^36^.

### Ethical approval and consent to participate

All subject investigations conformed to the principles outlined in the Declaration of Helsinki and have been performed with permission of the study protocol approved by the ethics committee of Salah Azaiz Institute (SAI), under same’s Ethics Committee registration number (#ISA/2019/04). All subjects were informed about the purposes of the study and consented in writing to participate in the study.

## Results

### Cohort

A total of 32 women with dense breast tissue and a positive screening mammogram were recruited before microbiopsy. Detailed clinicopathological characteristics of the cohort are described in Table 1. Blood samples were acquired from all subjects for cfDNA analysis. Tumor status was confirmed by the pathology report from nodule biopsy and subsequent ultrasound. A cohort of 12 subjects with no confirmed tumors were stratified as pos-neg (age: 42.00 ± 4.73, BMI: 31.29 ± 6.53); 33.33% had a family history of nonbreast cancer. The remaining 20 subjects with confirmed tumors, 11 in stage I and 9 in stage II (age: 43.50 ± 3.95, BMI: 29.76 ± 5.07), were placed in the pos-pos group; 70% had a family history of nonbreast cancer, and 15% had a breast cancer history. No significant differences were observed between groups concerning the clinicopathological parameters (Table 1).

**Table 1:** Participants' characteristics (Pos-pos and Pos-neg).

Parameters	Pos-pos N = 20 (%)	Pos-neg N = 12 (%)	Total N = 32 (%)	P ^1^
**Demographic**				
**Age** (*years*)^2^	43.50 ± 3.95	42.00 ± 4.73	42.94 ± 4.25	0.3673
**BMI**^2^	29.76 ± 5.07	31.29 ± 6.53	30.33 ± 5.60	0.4949
**Risk factors**				
Smoking (never/sometimes)	19/1	11/1	30/2	0.7061
Alcohol use (never/sometimes)	20/0	12/0	32/0	NA
**Clinical history**				
Hypertension	6 (30.00%)	1 (8.33%)	7 (21.88%)	0.1512
Hyperglycemia	2 (10.00%)	2 (16.67%)	4 (12.50%)	0.5809
Anemia	5 (25.00%)	2 (16.67%)	7 (21.88%)	0.5809
**Cancer family history**				
Other Cancer	11 (55.00%)	4 (33.33%)	15 (46.88%)	0.5153
Breast cancer	3 (15.00%)	0 (0.00%)	3 (9.38%)	0.1587
**TNM classification**				
I	11	NA	NA	NA
II	9	NA	NA	

*Pos-neg* Positive–negative subjects, *Pos-pos* Positive-positive subjects, *BMI* Body Mass Index, *TNM* Tumor, Nodes, Metastases according to Cancer (*AJCC* American Joint Committee on Cancer), *NA* Not Applicable.

^1^Pearson chi square (categorical variables), Student t-test (continuous variables), Value in bold is statistically significant < 0.05.

^2^Mean ± standard deviation.

### Tumor fraction estimation

**Figure 2:**
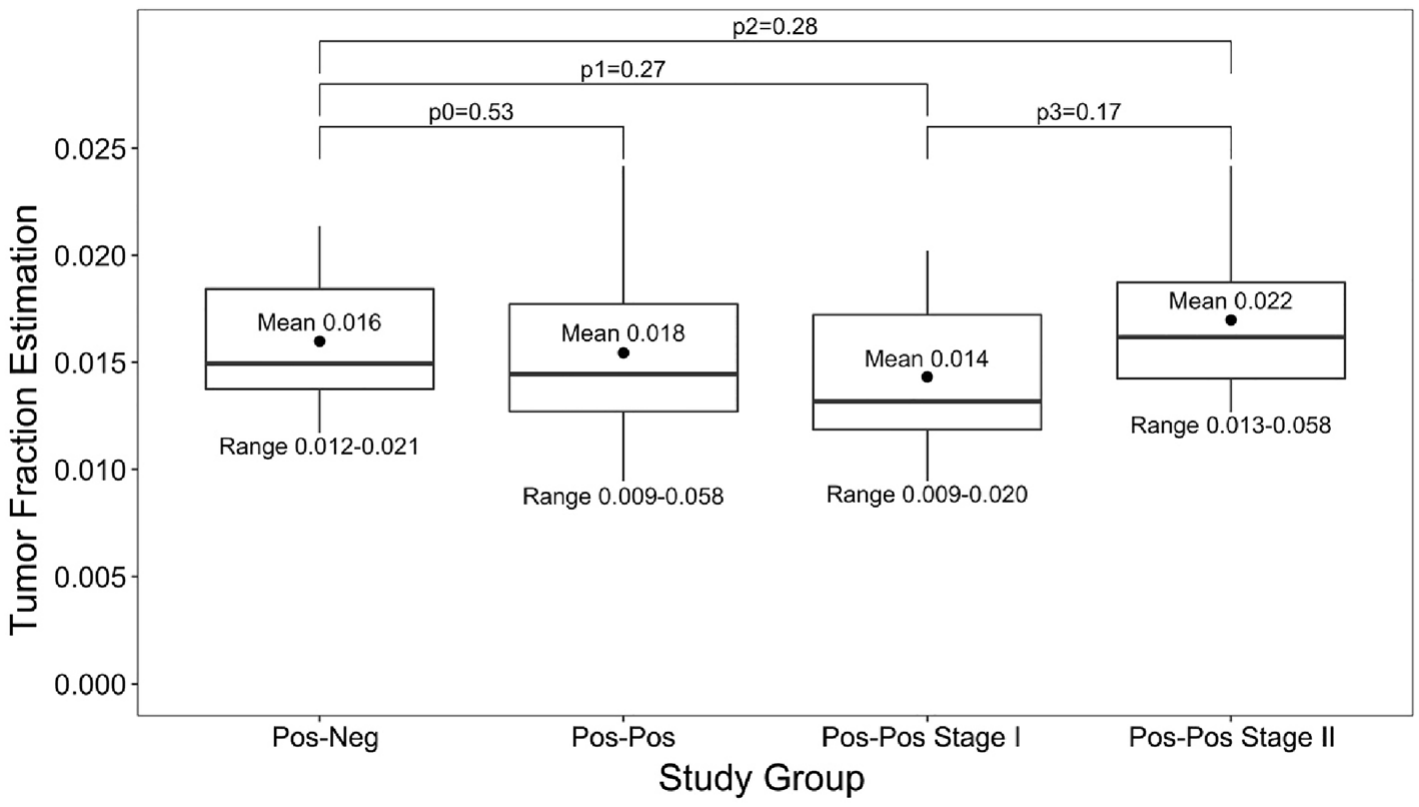
Distribution of tumor fraction estimation. *p0* Pos-neg vs. Pos-pos, *p1* Pos-neg vs. Pos-pos Stage I, *p2* Pos-neg vs. Pos-pos Stage II, *p3* Pos-pos Stage I vs. Pos-pos Stage II. p-value: Student t-test, *Pos-Neg* Positive–negative subjects, *Pos-Pos* Positive-positive subjects.

The level of tumor‐derived DNA in plasma at baseline (after the positive mammogram and before microbiopsy) was predicted. Subjects were first analyzed as one group and then stratified based on the biopsy pathological results into four groups (pos-neg subjects and pos-pos Stage I, pos-pos Stage II and all pos-pos subjects). The lower limit of sensitivity for detecting the presence of tumor or TF cutoff was set to 3%, as suggested by the authors of the ichorCNA software. For the pos-neg cohort, the mean TF was 0.016 (range 0.012–0.021), and for the all pos-pos group, the mean TF was 0.018 (range 0.009–0.058). The difference in mean TF between the two groups was not statistically significant (p0 = 0.53). The pos-pos TF range was wider, suggesting a larger deviance between TFs in the pos-neg group than in the pos-pos group. The mean TF for the pos-pos Stage I group was 0.014 (range 0.009–0.020) versus 0.022 (range 0.013–0.058) for the pos-pos stage II group; the differences between these groups and the pos-neg group were not significant (p1 = 0.27 and p2 = 0.28, respectively). The mean TF differences between the pos-pos Stage I and II groups was also not statistically significant (p3 = 0.17), although the pos-pos Stage II group had a larger mean TF and contained the only subject with a TF above the 3% cutoff (Fig. 2).

### CNAs and associated genes

**Figure 3:**
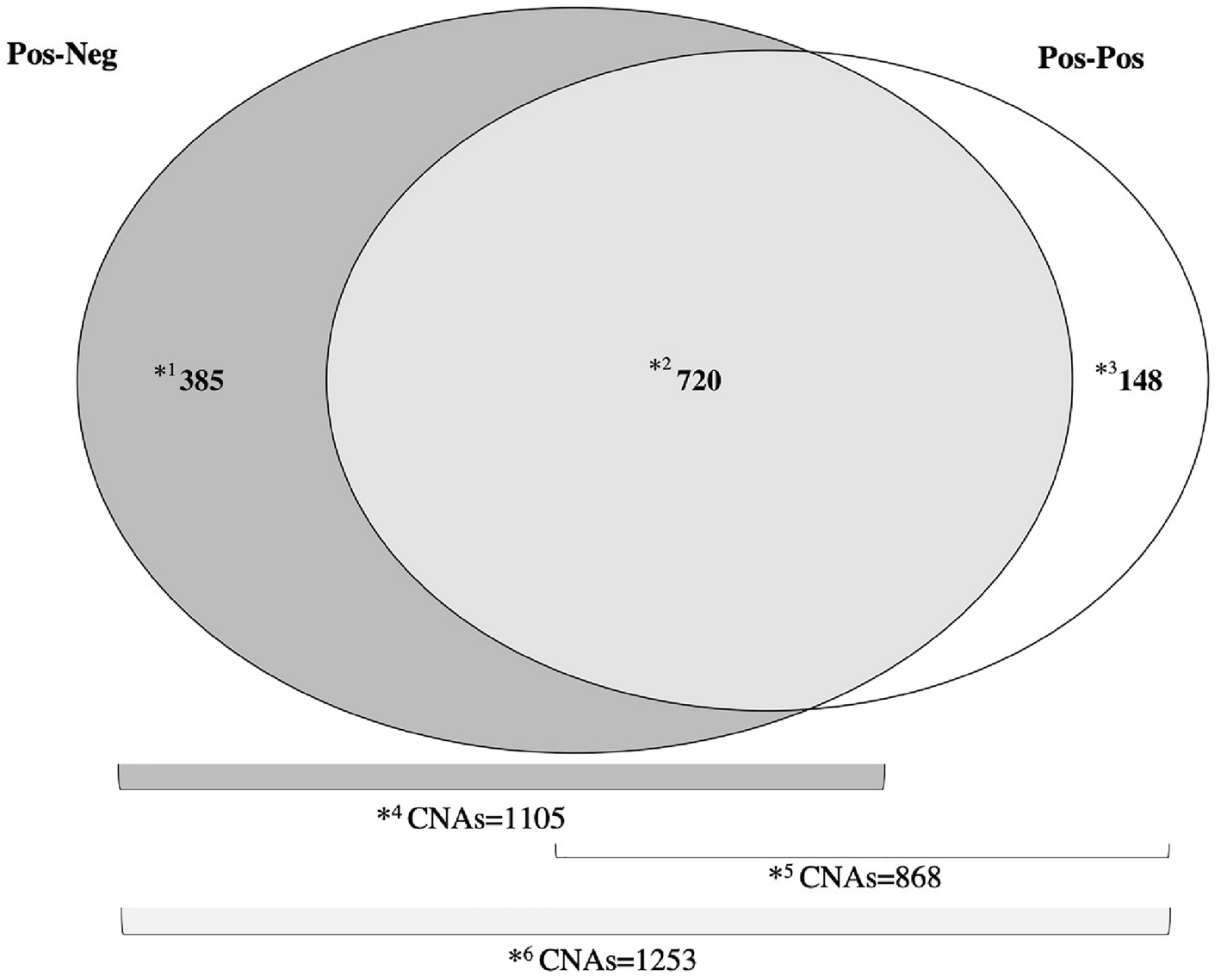
Diagram of the CNAs distribution in study groups. CNAs: Copy Number Alterations. *^1^: Exclusive CNAs detected in Pos-neg. *^2^: Shared CNAs between Pos-neg and Pos-pos. *^3^: Exclusive CNAs detected in Pos-pos. *^4^: Total CNAs for Pos-neg. *^5^: Total CNAs for Pos-pos. *^6^: Total CNAs detected in the study cohort (Pos-neg and Pos-pos). Pos-Neg: Positive–negative subjects; Pos-Pos: Positive-positive subjects.

CNA analysis detected a total of 1253 CNAs across all subjects, 1105 of which were in the pos-neg group and 868 in the pos-pos group. A total of 720 CNAs were shared by both groups, 385 found solely in the pos-neg group and 148 in the pos-neg group. The 1105 pos-neg CNAs were classified as gain (306), deletion (748) and amplification^51^. Of the 868 pos-pos CNAs, 382 were classified as gain, 435 as deletion and 51 as amplification (Fig. 3 and Table 2). Among the pos-neg subjects, chromosomes (Chr) 1 and 2 had the highest number of CNAs, 109 and 212, respectively, while for pos-pos cases, Chr 1 and 4 had 126 and 97 CNAs, respectively (Table 2). Of the 1253 total CNAs, 90 known overlapping oncogenes were identified; 15 were associated with CNAs found in both groups, 11 of which were previously described in cancers other than BC and 4 with a known association with BC. In addition, 49 deletion CNAs were detected in pos-neg subjects; 30 overlapped with genes previously described as associated with different cancers, 3 of which were previously associated with BC. On the other hand, 26 CNAs classified as gain were detected among the pos-pos subjects; 18 of these CNAs had a potential impact on genes that were previously described as associated with different cancers, 5 of which were described in BC (Table 3).

**Table 2:** Copy Number Alteration count for study subjects and stratified by subject’s group according to ichorCNA.

CNA filtering	CNA count						
All subjects	1253 (454 GAIN, 748 DEL, 51 AMP)						
Pos-neg	1105 (306 GAIN, 748 DEL, 51 AMP)						
Pos-pos	868 (382 GAIN, 435 DEL, 51 AMP)						
Total count	**GAIN**		**DEL**			**AMP**	
Subject segregation	**Pos-neg**	**Pos-pos**	**Pos-neg**		**Pos-pos**	**Pos-neg**	**Pos-pos**
Shared by at least 2 subjects in a group	200	355	563		435	51	51
Exclusive for a particular group	72	148	313		0	0	0
CNA location by chromosome	**Pos-neg**			**Pos-pos**			
CHR1	109			126			
CHR2	212			0			
CHR3	0			0			
CHR4	97			97			
CHR5	87			0			
CHR6	0			0			
CHR7	79			79			
CHR8	88			88			
CHR9	0			0			
CHR10	72			72			
CHR11	61			61			
CHR12	0			17			
CHR13	67			67			
CHR14	0			0			
CHR15	34			34			
CHR16	64			64			
CHR17	18			36			
CHR18	52			52			
CHR19	0			0			
CHR20	35			35			
CHR21	20			20			
CHR22	10			20			

*CNA* Copy Number Alteration, *Pos-neg* Positive–negative subjects, *Pos-pos* Positive-positive subjects, *CH* chromosome, *DEL* Deletion, *AMP* Amplification, *G1* Screening Subjects Group, *G2* Cases Group.

**Table 3:** Classification of Copy Number Alteration by gene and cancer impact according to study groups.

Copy number alteration						
Genes	Detected copy number alteration stratified by study groups					
	Genomic position	Location	Pos-neg	Pos-pos	CCGD classification	
					Cancer related	BC related
*JUN*	58780790_58784047	CHR1	DEL	Gain	Blood	**–**
*JAK1*	64833244_65000000	CHR1	DEL	Gain	Liver, Blood, Colorectal, Pancreatic	**–**
*NEGR1*	71395942_72000000	CHR1	DEL	Gain	Liver	**–**
*FUBP1*	77948404_77979086	CHR1	DEL	Gain	Liver, Blood, Colorectal, Pancreatic, Gastric	**–**
*RBM15*	110338505_110346681	CHR1	DEL	Gain	Liver, Blood, Colorectal	**–**
*VTCN1*	117143586_117210927	CHR1	DEL	Gain	Pancreatic	**–**
*DDR2*	162632463_162787405	CHR1	DEL	Gain	Sarcoma	**–**
*NUF2*	163321934_163355764	CHR1	DEL	Gain	**–**	**–**
*PBX1*	164559634_164851831	CHR1	DEL	Gain	Gastric	**–**
*TPR*	186311651_186375253	CHR1	DEL	Gain	Blood, Colorectal	**–**
*CDC73*	193121957_193254815	CHR1	DEL	Gain	Blood, Gastric	**–**
*PIK3C2B*	204422627_204490424	CHR1	DEL	Gain	Blood, Colorectal	**–**
*MDM4*	204516405_204558120	CHR1	DEL	Gain	**–**	**–**
*PGBD5*	230314489_230426332	CHR1	DEL	Gain	**–**	**–**
*FH*	241497602_241519761	CHR1	DEL	Gain	**–**	**–**
*PRDM16*	3069202_3438621	CHR1	NA	Gain	Blood, Colorectal, Pancreatic, Gastric	**–**
*CAMTA1*	7000001_7769706	CHR1	NA	Gain	Liver, Blood, Colorectal,	**–**
*SDHB*	17018721_17054170	CHR1	NA	Gain	**–**	**–**
*PAX7*	18630845_18748866	CHR1	NA	Gain	Colorectal	**–**
*CDC42*	22052708_22090807	CHR1	NA	Gain	Liver, Blood, Colorectal, Pancreatic	**–**
*STK40*	36339623_36385896	CHR1	NA	Gain	Liver, Blood, Colorectal	**–**
*CSF3R*	36466042_36483278	CHR1	NA	Gain	Blood, Colorectal	**–**
*RRAGC*	38838197_38859772	CHR1	NA	Gain	Liver, Blood, Gastric	**–**
*MPL*	43337848_43352772	CHR1	NA	Gain	Blood	**–**
*IGF1*	102395873_102480645	CHR12	NA	Gain	Liver, Pancreatic	**–**
*DTX1*	113057689_113098028	CHR12	NA	Gain	**–**	**–**
*TBX3*	114670254_114684175	CHR12	NA	Gain	**–**	**–**
***KSR2***	**117453011_117968990**	**CHR12**	**NA**	**Gain**	**–**	**BC**
*NCOR2*	124324414_124495252	CHR12	NA	Gain	Liver, Blood, Colorectal, Pancreatic, Skin	
***MAP2K4***	**12020876_12143828**	**CHR17**	**NA**	**Gain**	**Liver, Blood, Colorectal, Pancreatic**	**BC**
*CCT6B*	34927860_34961460	CHR17	NA	Gain	Blood	**–**
*COL1A1*	50183288_50201632	CHR17	NA	Gain	**–**	**–**
*HLF*	55264959_55325187	CHR17	NA	Gain	Liver	**–**
***MSI2***	**57256522_57684689**	**CHR17**	**NA**	**Gain**	**Liver, Blood, Pancreatic, Gastric, Thyroid**	**BC**
*GNA13*	65009288_65056740	CHR17	NA	Gain	Liver, Colorectal, Pancreatic	**–**
*AXIN2*	65528562_65561648	CHR17	NA	Gain	Colorectal, Lung, Endometrial, Bladder	**–**
***CANT1***	**79000001_79009817**	**CHR17**	**NA**	**Gain**	**–**	**BC**
*MN1*	27748276_27801756	CHR22	NA	Gain	**–**	**–**
*GTSE1*	46296869_46330810	CHR22	NA	Gain	**–**	**–**
*HLF*	55264959_55325187	CHR17	NA	Gain	Liver	
***MSI2***	**57256522_57684689**	**CHR17**	**NA**	**Gain**	**Liver, Blood, Pancreatic, Gastric, Thyroid**	**BC**
*MYCN*	15940549_15947004	CHR2	Gain	NA	**–**	**–**
*CENPA*	26786055_26794589	CHR2	Gain	NA	**–**	**–**
*PPP1CB*	28751747_28802930	CHR2	Gain	NA	Liver, Blood, Colorectal, Pancreatic	**–**
*ALK*	29192773_29921586	CHR2	Gain	NA	**–**	**–**
*YPEL5*	30146940_30160533	CHR2	Gain	NA	Liver	**–**
*EPAS1*	46297406_46386697	CHR2	Gain	NA	Liver, Blood	**–**
*FANCL*	58159246_58241350	CHR2	Gain	NA	**–**	**–**
*ETAA1*	67397321_67412089	CHR2	Gain	NA	**–**	**–**
*DCTN1*	74361153_74380355	CHR2	Gain	NA	Colorectal, Sarcoma	**–**
*INPP4A*	98444949_98581821	CHR2	Gain	NA	**–**	**–**
*SOS1*	39000001_39121051	CHR2	Gain	NA	Liver, Blood	**–**
*TET3*	74000001_74108176	CHR2	Gain	NA	Blood, Colorectal, Pancreatic, Gastric	**–**
*AFF3*	100000001_100106128	CHR2	Gain	NA	Colorectal, Blood	**–**
*CXCR4*	136114348_136116243	CHR2	DEL	NA	**–**	**–**
*LRP1B*	140231422_141000000	CHR2	DEL	NA	Gastric	**–**
***ACVR2A***	**147845028_147930822**	**CHR2**	**DEL**	**NA**	**Liver, Pancreatic, Colorectal, Gastric**	**BC**
*H3F3AP4*	174719907_174720318	CHR2	DEL	NA	**–**	**–**
*CHN1*	174799312_175000000	CHR2	DEL	NA	Blood	**–**
*HOXD13*	176092720_176095944	CHR2	DEL	NA	**–**	**–**
*HOXD11*	176104215_176109754	CHR2	DEL	NA	**–**	**–**
*NFE2L2*	177230307_177264727	CHR2	DEL	NA	Liver, Blood, Colorectal, Pancreatic	**–**
*PMS1*	189784380_189877629	CHR2	DEL	NA	**–**	**–**
*STAT1*	190969033_191000000	CHR2	DEL	NA	Blood	**–**
*STAT4*	191029575_191151590	CHR2	DEL	NA	Blood	**–**
*CREB1*	207529891_207603431	CHR2	DEL	NA	Blood, Sarcoma, Colorectal, Pancreatic, Gastric	**–**
*CPS1*	210477681_210678142	CHR2	DEL	NA	Liver, Colorectal	**–**
*ERBB4*	211375716_212000000	CHR2	DEL	NA	Liver	**–**
*IKZF2*	213005362_213151603	CHR2	DEL	NA	Blood	**–**
*BARD1*	214725645_214809683	CHR2	DEL	NA	**–**	**–**
*INHA*	219572309_219575711	CHR2	DEL	NA	**–**	**–**
*PAX3*	222200985_222298996	CHR2	DEL	NA	**–**	**–**
*ACSL3*	222861035_222944639	CHR2	DEL	NA	**–**	**–**
***CUL3***	**224470149_224585363**	**CHR2**	**DEL**	**NA**	**Lung, Blood, Sarcoma, Colorectal, Pancreatic, Gastric**	**BC**
*IRS1*	226731316_226799759	CHR2	DEL	NA	**–**	**–**
*ACKR3*	236569824_236582354	CHR2	DEL	NA	**–**	**–**
*HDAC4*	239048167_239400949	CHR2	DEL	NA	Blood, Colorectal	**–**
*DROSHA*	31400496_31532061	CHR5	DEL	NA	Liver	**–**
*LIFR*	38474962_38595404	CHR5	DEL	NA	Liver	**–**
*RICTOR*	38937919_39000000	CHR5	DEL	NA	Liver, Blood, Colorectal, Gastric	**–**
*MAP3K1*	56815548_56896152	CHR5	DEL	NA	Liver, Pancreatic, Colorectal, Skin, Thyroid	**–**
***PIK3R1***	**68215755_68301821**	**CHR5**	**DEL**	**NA**	**Liver, Colorectal, Pancreatic, Gastric, Thyroid**	**BC**
*ARHGEF28*	73626157_73941992	CHR5	DEL	NA	Colorectal, Pancreatic	**–**
*MEF2C*	88718240_88904257	CHR5	DEL	NA	Blood, Sarcoma, Skin	**–**
*ARHGAP26*	143000001_143229011	CHR5	DEL	NA	Blood, Liver, Colorectal	**–**
*CSF1R*	150053290_150113372	CHR5	DEL	NA	Blood, Sarcoma	**–**
*PDGFRB*	150113838_150155845	CHR5	DEL	NA	Blood	**–**
*CD74*	150400040_150412751	CHR5	DEL	NA	**–**	**–**
*EBF1*	158695919_159000000	CHR5	DEL	NA	Sarcoma	**–**
*GABRA6*	161685720_161702592	CHR5	DEL	NA	**–**	**–**

Bold indicates genes associated with BC.

*Pos-neg* Positive–negative subjects, *Pos-pos* Positive-positive subjects, *CHR* CHRomosome, *DEL* Deletion, *BC* Breast Cancer, *ID* Identification, *NA* Not Applicable, *CCGD* Candidate Cancer Gene Database.

### SNPs, indels and associated genes

A total of 1,583,400 variants, 1,282,284 SNPs, 47,693 multiple nucleotide polymorphisms (MNPs) and 253,423 Indels were identified across all subjects before MAF and CADD filtering, which subsequently yielded 1,467,158 (1,215,768 SNPs, 47,693 MNPs and 203,697 Indels) and 143,719 variants, respectively (134,929 SNPs, 2386 MNPs and 6404 Indels). Of these 143,719 variants, 9494 and 134,225 were identified as coding and noncoding variants, respectively. Of the 9494 total coding variants, 3196 were predicted to have deleterious impact; out of these variants, 2139 were exclusive to the pos-pos group, and 1048 were exclusive to the pos-neg group. Subsequently, 10 variants were identified as shared by at least 2 subjects, 6 for the pos-pos group and 4 for the pos-neg group. Of the 134,225 noncoding variants detected, 78,704 were exclusive to the pos-pos group, and 38,845 were exclusive to the pos-neg group. Thereafter, 3992 and 1144 variants were identified as shared by at least 2 subjects of each group, respectively. Functional annotation of the noncoding variants identified 7 intronic variants, 5 in pos-pos and 2 in pos-neg subjects, and 3 upstream and downstream variants, 2 in pos-pos and 1 in pos-neg subjects (Table 4). A final set of 25 variants overlapped with oncogenes. Eighteen variants were identified among the pos-pos subjects (6 coding and 12 non-coding), and 10 of these 18 variants were previously described to be associated with liver, blood, pancreatic and skin cancers; only one pos-pos variant, rs2884935, was found in a gene (KMT2C) associated with BC. Among the pos-neg subjects, 7 variants were related to oncogenes (4 coding and 3 non-coding), and 5 of these were associated with blood, colorectal and pancreatic cancers, but none were detected in the breast oncogenes (Table 5).

**Table 4:** Variants count with functional annotation of noncoding variants.

Variants filtering	Variant count	
FilterMutectCalls	Total: 1,583,400 (SNPs: 1,282,284; MNPs: 47,693; Indels: 253,423)	
< .01 AF 1000G ALL and non-TCGA ExAC ALL	1,467,158 (SNPs: 1,215,768; MNPs: 47,693; Indels: 203,697)	
CADD (SNPs) or CADD Indel (indels) Scaled Phred Score > 10	143,719 (SNPs: 134,929; MNPs: 2386; Indels: 6404)	
**Variant stratification**	**Coding Variants**	**Non-Coding Variants**
Total count	9494	134,225
Predicted deleterious by at least 3 of MutationTaster, PolyPhen V2, Provean and SIFT	3196	NA
Exclusive to a particular group	Total: (G1: 2139; G2: 1048)	Total: (G1: 78,704; G2: 38,845)
Shared by at least 2 subjects in same group	**Total: (G1: 6; G2: 4)**	Total: (G1: 3992; G2: 1144)
FunSeq2 Score > = 1.5	NA	**Total: (G1: 12; G2: 3)**
**Functional annotation of noncoding variants (FunSeq2 Score > = 1.5) according to ANNOVAR**		
**Variants annotation according to region hit from RefSeq**	**G1**	**G2**
Intergenic	2	0
Intronic	5	2
ncRNA_intronic	1	0
3’UTR	0	0
Upstream and Downstream	2	1
5’UTR5	2	0
ncRNA_exonic	0	0

Bold indicates final variant count after filtering.

*RefSeq* Reference sequence database, *ncRNA* non-coding transcript variant, *NA* Not Applicable, *ExAC* Exome aggregation consortium, *AF* Allele Frequency, *1000G* 1000 Genomes project for all individuals in this release, *CADD* Combined Annotation Dependent Depletion, *SNPs* Single Nucleotide Polymorphisms, *Indels* insertions/deletions, *MNPS* Multi-nucleotide Polymorphisms, *PolyPhen V2* PolyPhen Version 2, *G1* positive-positive subjects, *G2* positive–negative subjects, *SIFT* Sorting Intolerant From Tolerant, *PROVEAN* Protein Variation Effect Analyzer.

**Table 5:** Classification of detected variants by gene and cancer impact.

Genes	SNP ID	AF	Genomic structural	Functional annotation	Cancer related	BC related
Pos-pos						
*CNTN3*	*﻿ rs139142211*	0.0004	Coding	EX	**–**	**–**
*TMEM44*	*rs146561237*	NA	Coding	EX	**–**	**–**
*ANK2*	*﻿ rs776254819*	NA	Coding	EX	**–**	**–**
*SERAC1*	*rs757825963*	NA	Coding	EX	Blood	**–**
*DAGLB*	*﻿ rs766835420*	NA	Coding	EX	Blood, Colorectal	**–**
*TNC*	*﻿ rs376093344*	NA	Coding	EX	**–**	**–**
*MACF1*	*﻿NA*	NA	Noncoding	INT	Liver, Blood, Pancreatic	**–**
*BATF3*	*﻿NA*	NA	Noncoding	Upstream	**–**	**–**
*NVL*	*﻿NA*	NA	Noncoding	INT	Blood	**–**
*FBXW4*	*﻿ rs147494591*	0.0078	Noncoding	INT	Blood	**–**
*FANK1*	*﻿NA*	NA	Noncoding	INT	Colorectal	**–**
*KCTD4*	*﻿NA*	NA	Noncoding	5’UTR	Colorectal	**–**
*SHF*	*﻿NA*	NA	Noncoding	Upstream	**–**	**–**
*CAVIN1; ATP6V0A1*	*rs190711126*	0.0004	Noncoding	Intergenic	Blood, Colorectal, Pancreatic	**–**
*HIF3A*	*﻿NA*	NA	Noncoding	5’UTR	**–**	**–**
*LOC101927050; LOC654342*	*﻿ rs11883680*	NA	Noncoding	Intergenic	**–**	**–**
*ZBTB20-AS1*	*rs114892760*	0.0032	Noncoding	ncRNA_intronic	Liver, Blood, Pancreatic, Skin	**–**
***KMT2C***	***﻿ rs2884935***	**NA**	**Noncoding**	**INT**	**Liver, Blood, Pancreatic, Colorectal, Gastric**	**Breast**
Pos-neg						
*SNIP1*	*rs202020647*	0.0002	Coding	EX	Colorectal	**–**
*ATP2A1*	*rs769732457*	NA	Coding	EX		**–**
*TBC1D10B*	*﻿ rs145571848*	NA	Coding	EX	Blood, Colorectal	**–**
*EVPL*	*rs201833287*	0.0002	Coding	EX	**–**	**–**
*PANK1*	*﻿NA*	NA	Noncoding	Upstream	Liver, Blood	**–**
*PRKCA*	*rs139323901*	0.003	Noncoding	INT	Blood, Colorectal, Pancreatic, Gastric	**–**
*RUNX2; SUPT3H*	*NA*	NA	Noncoding	INT	Blood	**–**

Bold indicates genes associated with BC.

*AF* 1000G Phase 3 all population Allele Frequency, *Column in bold* variant previously described as associated with cancer, *BC* Breast Cancer, *SNP* Single Nucleotide Polymorphism**, ***Pos-neg* positive–negative subjects, *ID* Identification, *Pos-pos* positive-positive subjects, *rs* reference SNP, *INT* intronic, *EX* EXonic, *NA* Not Applicable, *G* Group, *Cancer related* according to Candidate Cancer Gene Database.

Significant values are in bold.

## Discussion

Multiple studies have demonstrated the significance of a noninvasive ctDNA variant testing biopsy for the early detection of solid tumors and subsequent improved outcomes^37^, therapy management^38^, response assessment^39^, and tumor resistance^40^. Short-fragment, low tumor-fraction cfDNA testing presents a challenge to early detection efforts, however. These fragments were largely investigated in clinical applications related to treatment prediction, relapse, and drug resistance^41^. Most previous studies focused on cfDNA levels as a predictive biomarker for therapeutic response in solid cancers^42^. Recently, a large-scale study based on cfDNA concentration showed that variation in the cfDNA level in plasma is not related to patient outcome and thus suggested that cfDNA concentration could not serve as a reliable biomarker for cancer management^43^. However, investigating cfDNA molecular profiles remains a viable opportunity for evaluating their relationship in detecting and characterizing the patient’s cancer status. In this study, we report a combined analysis of cfDNA whole-genome profiles between subjects with positive mammograms and biopsies versus subjects with positive mammograms and negative biopsies and suggest the possible role of these differences in the early detection of BC and subsequent clinical diagnosis, precision treatment protocols, and hopefully improved outcomes.

According to our assessment of previous research, our study is the first to examine and propose a full ctDNA analysis, including CNA and SNP/Indel detection and characterization, for identifying breast tumors in dense tissue subjects before mammogram identification. We assert that such an approach, when demonstrated to be robust, could serve as a precision oncology application in early BC detection.

In this study, the mean TF (0.016 and 0.018 for the pos-neg and pos-pos groups, respectively) was lower than the 3% recommended TF cutoff. The low TFs obtained in this study may be related to the low sensitivity in detecting the presence of ctDNA in our sequenced data^19^. However, the TF ranges were larger in the pos-pos group than in the pos-neg group and thus are possibly a different indicator of the presence of cancer than the TF alone. In addition, a higher TF was found in pos-pos stage II than in pos-pos stage I, suggesting that the ctDNA fraction increases as a function of tumor progression. These results support the interpretation that the isolated DNA fragments were ctDNA, an interpretation consistent with previous liquid biomarker studies investigating cfDNA as an early detection and prognosis biomarker in BC^44^. Other studies have demonstrated the reliability of ctDNA biomarkers for cancer therapeutic decision-making, evaluating patients’ resistance to treatment^45,46^, and tracking tumor progression during and after therapy^47,48^. The results of this study identified deletion and gain CNAs exclusively found in pos-neg subjects that overlapped across 11 known oncogenes. Three of these genes, JAK1, FUBP1, and RBM15, are all associated with liver, blood, colorectal and pancreatic cancers; three, TPR, CDC73 and PIK3C2B are all associated with blood and colorectal cancers; and five, JUN, NEGR1, VTCN1, DDR2 and PBX1, are associated with blood, liver, pancreatic, sarcoma and gastric cancer, respectively. In addition, among the pos-neg subjects, three exclusive deletion CNAs overlapped with the ACVR2A, CUL3 and PIK3R1 oncogenes, which are associated with BC. Among the pos-pos subjects, five exclusive gain CNAs overlapped with the KSR2, MAP2K4, MSI2, CANT1 and MSI2 oncogenes, all previously associated with BC (Table 3). Differences in the detected deletion and gain CNAs associated with pos-neg and pos-pos subjects may be related to epigenetic modifications and their impact on somatic alterations leading to oncogenesis and tumor growth^49^. The precise differences in nucleosome positioning between tumor and normal cells have been described as actively involved in the footprints of transcription factors associated with oncogenesis detectable in cfDNA fragments^50^. The nuclear architecture responsible for gene structure and expression has been correlated with cfDNA nucleosome occupancies, suggesting the potential for the early-stage detection of cancer cells^51^. Recently, these same nucleosome footprints identified cell types shedding cfDNA whose molecular profile suggested involvement in multiple pathological states, including cancer^52^. cfDNA profiling was also found to be informative of tumor localization and progression^53^. Differential release of cfDNA was also correlated with tumor heterogeneity among patients diagnosed with similar cancers and thus could be a promising biomarker of therapy management^54^. The collective evidence from the current and previous studies suggests that CNAs previously described in breast tissue coupled to their presence in a ctDNA-based biopsy may play an important role in the early detection and diagnosis of BC. The SNP and Indel results identified 10 functionally important variants in the pos-pos subjects previously associated with cancer. One variant, rs757825963, was located in SERAC1, a known BC risk factor. In addition, SERAC1 is also associated with leukopenia^55^, and increased expression of SERAC1 has been correlated with BC risk^56^. SERAC1 also has a strong interaction with multiple splicing factors (hnRNP A3, hnRNP J, hnRNP G, FMRP, Fox-2) in the context of cancer prognosis and development^57^. The clear and important role of SERAC1 in splicing events suggests a likely role as an early detection liquid biopsy biomarker when coupled to the role of cfDNA variants associated with dysregulation related to epigenetics. Another identified variant, rs147494591, found in FBXW4, which encodes for the F-box proteins that are involved in biological processes such as cell growth, division, development, differentiation, survival and death^58^, suggests another possible molecular biomarker for early BC detection. Previous studies found that decreased expression of FBXW4 was correlated with poor survival among non-small-cell lung cancer patients^59^. A recent study showed that downregulation of FBXW4 favored colorectal tumor relapse and limited the survival range^60^. Together with the results of this study, these previous study findings suggest that FBXW4 may be an important prognostic indicator in oncology. Pos-pos subject variants identified in NVL suggest a role in the dysregulation of telomere function, possibly initiating breast tumor development. The depletion role of NVL was strongly associated with lower hTERT, associated with decreased telomerase activity in multiple pathogeneses^61^. Two exclusively pos-pos variants found in known BC risk-associated genes (FANK1 and KCTD4) suggest further pos-pos cfDNA somatic association with BC risk. FANK1 was recently identified as a novel binding partner in mammalian cells that prevents the proteasome degradation of polyubiquitinated FANK1, which leads to the activation of the AP-1 signaling pathway and the induction of tumor cell apoptosis^62^. KCTD4 was reported as a tumor suppressor gene associated with insertional mutagenesis for leukemia or lymphoma development in insertional mutagenesis in a mouse model study^63^. The deregulation of both FANK1 and KCTD4 may be a consequence of the observed somatic variants, thus suggesting another association with tumor development and their use as an early detection biomarker in a cfDNA-based assay. The two pos-pos–associated variants (rs766835420 and rs190711126), located in DAGLB and CAVIN1/ATP6V0A1, respectively, were positively associated with BC. SNPs of DAGLB have been correlated with increased DAGLB expression in stomach tissues and were also significantly elevated in gastric tumors compared to adjacent tissues, thus confirming the potential of DAGLB as a susceptibility gene for gastric cancer^64^. Loss of stromal CAVIN1 expression negates the ability of stromal cells to sequester lipids and is associated with the upregulation of inflammatory factors such as cytokines and their receptors, matrix metalloproteinases, and markers for CAFs^65^. Deregulation of any inflammatory microenvironment factors, such as those seen in CAVINI, promotes aggressive cancer phenotypes, thus supporting the critical function of CAVINI in the stromal component in tumorigenesis and suggesting a metastasis-suppressing role for this gene^66^. Any deleterious variant appearing in CAVIN1 will likely contribute to lower CAVINI expression and loss of stromal cell function, suggesting a role in breast cancer genesis and tumor development. Other deleterious pos-pos variants found in MACF1 and ZBTB20-AS1 align with earlier studies showing that MACF1 mutations detected in tissue-specific genomes are responsible for function dysregulation associated with cancer^67^, and a correlation study found that key ZBTB20-AS1 lncRNAs are associated with colon tumor staging and likely tumor progression^68^. Finally, a pos-pos exclusive variant was associated with KMT2C, a known BC risk factor. In addition, KMT2C is the gene with the highest mutation count predominantly found in BC, with some mutations associated with chromatin function, affecting transcription mechanisms identified in breast tumor development^69^. KMT2C mutations were also shown to be key to ERα regulation, which can lead to hormone-driven breast cancer cell proliferation^70^. In summary, the somatic variants found in the pos-pos cases investigated in this study present a rich and highly associated set of potential biomarkers shown to affect key molecular mechanisms important to oncogenesis (and its suppression) and therefore may be putative biomarkers for early BC detection.

Concerning the pos-neg screening group, 6 oncogenes were identified as containing exclusive variants: SNIP1, TBC1D10B, PRKCA, RUNX2 and SUPT3H. PRKCA has been previously identified as associated with BC and encodes a calcium-dependent protein kinase involved in multiple biological functions, including calcium ion transport, exocytosis, cell growth, and proliferation^71^. PRKCA is also a central signaling node and coinhibitor of the ESR1, mTORC1, and HDAC genes known to suppress breast cancer^72^. The collective evidence suggests that PRKCA is an important candidate for breast carcinoma stem cell management^73^. Two hypotheses suggest a role for PRKCA somatic variants in the absence of cancer in pos-neg subjects. First, these variants may have a protective effect against BC oncogenesis via the modulation of PRKCA expression, thus delaying if not stopping tumor development and growth.

Despite the notable results, there are limitations to be acknowledged. This is a small subject study, and a large cohort study must follow to validate these results and thereby challenge the robustness of the proposed biomarkers. Additionally, it is important that an additional study be performed with healthy control subjects (neg-neg) to test for any BC-associated cfDNA variants. These studies should also include normal tissue (from all subjects) and tumor tissue samples (from pos-pos cases) to validate the cfDNA profile against the tumor profile, thus confirming that cfDNA is actually ctDNA. TF levels must also be tested against presence and staging to further validate the use of TF range and low TF to confirm tumor presence and absence. Some detected variants in the pos-pos case group were previously detected in non-BC tumors. This result raises the possibility that such ctDNA variations may be present due to genome disorder, suggesting that these may not be valid biomarkers for BC.

## Conclusions

Early breast cancer detection is of paramount importance in managing the most common cancer worldwide. Any bioassay suggested to be a robust test of early BC must be precise, repeatable, inexpensive and preferably noninvasive to replace the standard mammogram-biopsy protocol for BC diagnosis, but at this time, no such bioassay exists. Studies such as this in dense tissue subjects demonstrate promising evidence that a low-TF (thus providing early detection), noninvasive, robust bioassay may be available through cfDNA molecular testing. The presented results and suggestion are the first to describe a coupled analysis of CNA and SNP/Indel identification using cfDNA profiles for breast cancer early detection. Before these promising results can be used in the development of a panel of biomarkers for a biopsy, further understanding of early breast tumor biology and of the mechanisms that lead to tumor progression, is greatly needed to identify the molecular biomarkers to be used with such a highly informative assay. The molecular profiling and analysis workflow performed in this study on cfDNA taken from early screened and confirmed BC subjects presents promising results contributing to the knowledge required to create such a liquid biopsy test. Further investigations building on this are needed to confirm the results of this study, test the putative cfDNA molecular biomarkers and confirm their validity for inclusion in an early BC detection bioassay. In this way, these biomarkers could can contribute to significant improvements in BC diagnosis and therefore improved treatment optimization and subsequent outcomes to reduce the devastating incidence and mortality of breast cancer.

## Data Availability

The datasets used and analyzed during the current study are available from the corresponding author on reasonable request.

## Abbreviations

cfDNA: Cell-free DNA
BC: Breast cancer
CNAs: Copy number alterations
ctDNA: Circulating tumor DNA
NGS: Next-generation sequencing
SNPs: Single nucleotide polymorphisms
Indels: Insertions/deletions
MM: MagMAX
TF: Tumor fraction
MAF: Minor allele frequency
MNPs: Multiple nucleotide polymorphisms
